# Does physical activity change predict functional recovery in low back pain? Protocol for a prospective cohort study

**DOI:** 10.1186/1471-2474-10-136

**Published:** 2009-11-06

**Authors:** Paul Hendrick, Stephan Milosavljevic, Melanie L Bell, Leigh Hale, Deirdre A Hurley, Suzanne M McDonough, Markus Melloh, David G Baxter

**Affiliations:** 1 Centre for Physiotherapy Research, School of Physiotherapy, University of Otago, Dunedin, New Zealand; 2 Department of Preventive and Social Medicine, Dunedin School of Medicine, University of Otago, Dunedin, New Zealand; 3 School of Physiotherapy and Performance Science, College of Life Sciences, University College Dublin, Ireland; 4 Health & Rehabilitation Sciences Research Institute, School of Health Sciences, University of Ulster, Northern Ireland; 5 Section of Orthopaedic Surgery, Department of Medical and Surgical Sciences, Dunedin School of Medicine, University of Otago, Dunedin, New Zealand

## Abstract

**Background:**

Activity advice and prescription are commonly used in the management of low back pain (LBP). Although there is evidence for advising patients with LBP to remain active, facilitating both recovery and return to work, to date no research has assessed whether objective measurements of free living physical activity (PA) can predict outcome, recovery and course of LBP.

**Methods:**

An observational longitudinal study will investigate PA levels in a cohort of community-dwelling working age adults with acute and sub-acute LBP. Each participant's PA level, functional status, mood, fear avoidance behaviours, and levels of pain, psychological distress and occupational activity will be measured on three occasions during for 1 week periods at baseline, 3 months, and 1 year. Physical activity levels will be measured by self report, RT3 triaxial accelerometer, and activity recall questionnaires. The primary outcome measure of functional recovery will be the Roland Morris Disability Questionnaire (RMDQ). Free living PA levels and changes in functional status will be quantified in order to look at predictive relationships between levels and changes in free living PA and functional recovery in a LBP population.

**Discussion:**

This research will investigate levels and changes in activity levels of an acute LBP cohort and the predictive relationship to LBP recovery. The results will assess whether occupational, psychological and behavioural factors affect the relationship between free living PA and LBP recovery. Results from this research will help to determine the strength of evidence supporting international guidelines that recommend restoration of normal activity in managing LBP.

**Trial registration:**

[Clinical Trial Registration Number, ACTRN12609000282280]

## Background

Low back pain (LBP) is a common [1] costly [2] and at times disabling condition [3] with a high prevalence across a range of occupational settings [4-7]. Although LBP mostly settles within 3-4 weeks [8] of onset, a significant proportion will either not resolve or will recur [9]. The direct and indirect healthcare costs associated with chronic LBP and resultant disability and work absence are considerable [10]. In an attempt to reduce costs and facilitate return to work, national and international LBP guidelines [11,12] recommend restoration of normal activity as an integral part of the management of acute and sub-acute back pain, and occupational health guidelines recommend and encourage an early return to work [13]. Advice to stay active as an adjunct to exercise produced more favourable results then exercise alone when included in standard care for patients with acute LBP [14] and there is strong evidence for supporting early mobilisation and activity in the treatment of acute LBP [15,16].

Currently there is limited evidence for physical activity (PA) facilitating prognosis of occupational LBP [17-19]. One study found that regular exercise outside of work tended to protect against recurrence of work-related LBP [19], and another that leisure time PA levels were predictive of return to work in patients who had undergone a light mobilization program after initial LBP sick leave [17]. Storheim [18] also found a significant positive relationship between higher fitness levels and return to work in patients with chronic LBP. A number of studies have explored relationships between PA and risk of developing occupational LBP [20-22], or looked for relationships between pre-injury activity and LBP outcome [23]. A recent systematic review did not identify activity level as a predictor for return to work in patients with acute LBP [24], however none of the included studies objectively and prospectively measured PA, and its relationship to outcome.

Cross sectional studies provide little evidence for a direct link between PA and LBP outcomes [25-27] and prospective cohort studies investigating the potential relationships between PA levels and LBP show mixed results [28-31]. Bousema [29] employed an accelerometer to measure PA at two time points over a 1 year period and reported no difference in PA level changes between groups classified as "recovered LBP" and "non-recovered LBP". Similarly, Leonhardt [30], using an activity recall questionnaire, found that development of chronic pain had no influence on the total energy expenditure at six months in a mixed cohort of patients with acute and chronic LBP. Hurwitz [28] reported that both the cross sectional and longitudinal odds ratio for back disability were significantly reduced in those who had the greatest levels of leisure time PA levels. However, Mortimer [31] found that non-specific regular exercise (measured with a recall questionnaire) did not seem to improve LBP outcomes at 5 year follow-up. Thus evidence for a relationship between PA levels in free living and functional recovery in patients with LBP appears to be equivocal.

Research priorities in LBP and the prevention of work-related musculoskeletal disorders recommend prospective studies to monitor and assess the natural course of LBP over time, and in particular to look at factors that are predictors for chronicity and search for effective preventive measures [32,33]. Dose response relationships between activity levels, including types, frequencies and duration should also be explored using validated functional outcome measures [34,35].

Accurate assessment of the amount and intensity of PA in daily life is considered important due to the strong relationship between PA levels, health and disability [36,37]. However, it is recognised that the measurement and capture of the various dimensions of PA in free living is problematic [38]. Variance and fluctuations within normal free living activity levels, [39,40] and debate regarding measurement tools and time required to accurately portray and reliably measure free living PA, [41,42] mean that there is a lack of consensus on an optimal approach to measure free living activity. However, recommendations suggest a repeated measures (longitudinal) design that includes an objective measure of PA [43,44] will likely provide the most accurate method of estimating PA in free living [45]. To date no studies have prospectively employed an objective measure of PA to investigate the predictive relationship between PA in free living and a validated LBP outcome in a cohort of patients with acute LBP.

Accelerometers provide an objective tool for the assessment of PA in free living populations over periods long enough to be representative for normal daily life [44]. Triaxial accelerometers have been used to measure and quantify PA within several patient populations [46-48]. The RT3 triaxial accelerometer provides a valid and reliable measure of PA [49-51] and has been previously utilised as a measure of PA change within defined patient populations [52,53].

## Methods

### Research objectives

1. To investigate the predictive relationship between change in objectively measured PA levels in a cohort of patients with acute (< 6 weeks' duration) LBP [54] from baseline to 3 months, and change in functional outcome at 3 months and recovery over a 1 year period.

2. To assess the effect of occupation and occupational activity levels, personal factors, pain levels, functional status and psychosocial profile on the relationship between PA levels and LBP outcomes at 3 months and at 1 year.

3. To determine the relationship between restoration of "normal" levels of PA as considered by the patient and functional recovery.

### Hypotheses

Three specific hypotheses will be tested:

1. Positive changes in PA levels of participants with acute LBP (from baseline to 3 months) are a positive predictor of recovery (defined from change score in *Roland Morris Disability Questionnaire*) [54] at 3 months and at 1 year;

2. Psychosocial factors including levels of fear avoidance beliefs, depression and anxiety and occupational factors including types of occupation and levels of manual or sedentary work act as confounders in the relationship between activity change and the course of LBP over a 1 year period;

3. Restoration of "normal" levels of PA at baseline and at 3 months is a positive predictor of functional recovery over a 1 year period.

### Study design

A cohort study recruiting patients by public advertising: this will include local newspapers, public notice boards, posters, mail-outs to local physiotherapy clinics in the urban and sub-urban environment of a city in New Zealand, as well as email notification of university staff and students at the University of Otago, Dunedin. All participants who are interested in the study will be encouraged to contact the principal investigator (PI) via telephone or e-mail. Recruitment will take place over a one year period. Assessment and monitoring of each participant's activity levels will take place after attending their physiotherapist for acute LBP treatment with follow-up assessments and activity measurements at 3 and 12 months.

The study protocol has been approved by the Lower South Regional Ethics Committee (LRS/07/11/043) and the Ngâi Tahu Research Committee following Maöri consultation.

### Inclusion and exclusion criteria

To be eligible to take part in the study, participants must fulfil the following criteria:

1) Have an episode of LBP of 6 weeks or less, proceeded by at least 3 months of relative freedom from symptoms. These inclusion criteria will effectively exclude the chronic LBP population (defined as symptoms exceeding 3 months) [55].

2) Be between the ages of 18 and 65 years (working age population).

3) Be English speaking and able to provide informed consent to PA monitoring and follow up for 12 weeks.

4) Have no other pre-existing conditions which limit their mobility of PA levels.

5) Be receiving physiotherapy treatment for this current episode of acute LBP. This threshold was chosen to make results generalizable to this specific patient population.

6) Have a minimal score of 4 on the RMDQ. This score will allow for the detection of the smallest clinically important change [55].

The following exclusion criteria will be investigated at an initial screening interview by the PI prior to recruitment:

1) Serious or systemic spinal pathologies including persistent or progressive neurological deficit, intractable pain, spinal surgery, or inflammatory disorders as assessed by their health practitioner or by screening questions from the co-investigator.

2) Any history of current or past medical problems (other than LBP) which prevent participants from undertaking usual day-to-day activities.

### Clinical screening

The PI will arrange a suitable appointment to determine eligibility for inclusion into the study based on a screening questionnaire administered via telephone which will search for evidence of serious spinal pathology and discuss the study protocol and requirements of the participant. The screening questionnaire is adapted from the New Zealand Accident Compensation Corporation (ACC) guidelines [12] which have been designed to detect any potentially significant symptoms of serious spinal pathology. Evidence for such a disorder will exclude the participant from the study.

### Research protocol and timetable

At an initial visit each participant's weight, height, age, sex, occupation and ethnicity will be recorded. Occupation coding will be carried out according to Australian and New Zealand Standard Classification of Occupations [56]. Also recorded, whether the participant is working or off-work due to the current LBP episode, the number of days of the week and total average hours that each participant works, and whether the participant considers their work to be either manual or sedentary. This occupational information will be used to provide a baseline descriptive account of the group, and also included as a potential confounder in the relationship between PA and functional recovery.

Participants will complete a number of validated functional LBP outcome measures at baseline [57]. The primary outcome measure is the RMDQ, accepted as a valid and sensitive measure of condition-specific functional outcome in LBP populations [58], and considered as the preferred instrument for assessing change in function over time in LBP [59]. Secondary outcomes will be the *Visual Analogue Scale *(VAS) measure of pain over the past 7 days which has been shown to be a valid and reliable clinical measure of pain in LBP populations [60] and a specific activity question developed for this study which asks the participant whether they have returned to full "normal' activities since the episode of low back pain (Y/N).

Other measures will investigate for potential confounding factors in the relationship between PA and functional recovery (RMDQ). These are measurements of depression, anxiety, emotional distress and fear avoidance and occupational activity levels and intensity. The *Baecke Questionnaire *(BPAQ) will be used to record the level of PA in the month prior to the current episode of LBP and activity levels at the 1 year point. The BPAQ is a reliable and valid measure of activity in both free living and LBP populations [61,62]. This questionnaire is divided into 3 sections which are individually scored: leisure time index, work index and sports index. The BPAQ work index score will thus provide a measure of the relative intensity of work activities [63] and occupational activity levels [64]. Fear avoidance beliefs will be measured with the *Fear-Avoidance Beliefs Questionnaire *(FABQ); a reliable measure of pain-related fear in acute LBP [65] and a valid measure for functional disability in acute and chronic LBP populations [66,67]. The 12-item *General Health Questionnaire *(GHQ12) will be used as a validated measure for evaluating levels of anxiety and depression in the general population [68].

### Physical activity measurement

All RT3 monitors will undergo testing prior to field use as part of the standardised protocol recommended when employing accelerometry measurement [69,70]. Previous research has shown high levels intra-monitor variability [49] and therefore each participant will be required to use the same RT3 monitor at baseline and 3 months. Field practice of monitor use and monitor placement will be standardised by asking the participants to wear the RT3 monitor on the right hip for all waking hours; to report wear times and reason for removal; to note the days that they work; and also to report sleep patterns [70] and hourly activities in an activity diary over the week [53].

The dimensions and specifics of the RT3 have been reported previously [49]. The RT3 triaxial accelerometer stores accumulated activity counts, derived from the three axes ([X^2 ^+ Y^2 ^+ Z^2^]^0.5^) to calculate a summed VM. Mode 4 will be employed for this study, which stores accumulated activity counts every second and calculates an average VM for each one-minute epoch over the 7 days of monitoring. Previous research has validated the use of RT3 VM counts as a measure of free living activity and energy expenditure [50].

Participants will be contacted twice during this week by text and/or phone to improve compliance in wearing the RT3, recording activity in the activity dairy, and to address any problems the participants might be having with either the RT3 or in using the activity diary. At the completion of the week the RT3 data will be downloaded to a portable computer and each participant will complete a 7-day recall questionnaire (7D-PAR) [71,72]. The 7D-PAR provides an estimate of the average total daily energy expenditure (TDEE) and physical activity energy expenditure (PAEE) for each participant.

All participants will be sent a reminder letter between the first and second period of monitoring; further contact will be made approximately 1 week prior to their scheduled date for re-monitoring at the 3-month point. Each participant will then repeat the activity monitoring procedure as per the baseline. At completion of week 12 each participant will be asked to complete the RMDQ, VAS and simple activity question and in addition an RT3 utility questionnaire developed for this study. This will assess any specific utility issues of PA measurement within this patient population.

**Figure 1:**
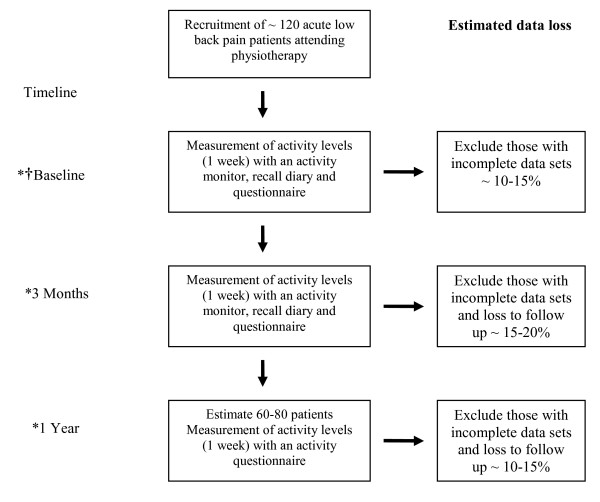
**Outline of study protocol**. *At each time point data on low back pain and functional recovery will be collected. † At baseline data on occupational, psychological and behavioural factors collected

At 1 year each participant will be sent the following questionnaires: VAS, FABQ, RMDQ, the BPAQ, GHQ12 and a modified Nordic LBP Questionnaire [73] with a self return envelope. The Nordic LBP Questionnaire has been previously employed as a measure of LBP recovery in an occupational setting [74] and as a measure of incident LBP, for assessing the associations between physical activity and incidence of LBP [75]. The BPAQ will be used to record current levels of PA at the 1 year point. The estimated duration of the study will be approximately two years to complete recruitment and 1 year follow-up (Figure 1).

### Data management

Following collection, data will be checked for accuracy and completeness and will involve:

• Download of all accelerometry data via StayHealthy^tm ^software into an Excel database.

• A visual review of all accelerometer data to determine the number of days of accelerometer data and to determine whether this satisfies the research protocol criteria.

The downloaded accelerometry data will be checked to ensure that the number of days of accelerometer data matched the protocol, to ascertain sleep times, and to identify possible RT3 malfunctions [76]. Data will then be scanned for nonworn periods. Such data will be set to 'missing' [70]. The data will then be separated into weekdays and weekend days. The sum of RT3 activity counts for each day will be calculated as well as the total number of hours of activity data collected each day. Total weekly activity count will then be divided by the total number of hours worn from each valid day of data collection [76]. The total RT3 score for the 7 days of activity monitoring will be expressed as average VM counts/hour/week. Estimates of data loss and the reasons for any physical activity data loss will also be investigated and a sensitivity analysis performed to investigate the effect of wear time on the relationship between activity change and RMDQ [77].

### Sample size and statistical power

We calculated sample size based upon the detection of a difference in change in RMDQ from baseline to three months in two groups classified as high changers in PA and low changers, as defined by the upper quartile of change in PA. This is a simplification of the actual analysis, but is conservative. The sample size is calculated for 80% power at a two-sided type I error rate of 0.05. Assuming a standard deviation of change in RMDQ score of 5.4 over a 3 month period, [78,79] the detection of a clinically meaningful change of 4 points in the RMDQ score from week 1 to week 12 [55,80], and an assumption of unequal group sizes, the required sample size is 65 participants. Previous research employing the RT3 in free living has found loss of data due to technical issues including monitor malfunction to be a significant issue [81,82]. Therefore a reasonably high attrition rate and potential dropout rate of 40-50% will be used over the 3 time points and this requires data from approximately 120 participants to be collected (Figure 1).

## Analyses

Multiple linear regression will be used to investigate the associations of the main predictor variable, change in RT3 VM counts/hour/week (ΔRT3) from baseline to 3 months, with the main outcome variable change in functional outcome (ΔRMDQ). The change score will be calculated by subtracting each participant's 3 month RMDQ score from their baseline RMDQ score. Unadjusted and adjusted analyses will be performed. Adjusted analyses will include the following variables, shown to be putatively associated with both PA and functional recovery in LBP: age, gender, occupation, baseline pain level (pain questionnaire), functional status (baseline RMDQ) and baseline measurements of depression, anxiety, emotional distress and fear avoidance (GHQ12 and FABQ). We plan to analyze each of the explanatory variables univariately initially to assess the relationship with the dependant variable (RMDQ change). We will include factors in the model which in univariate analyses have a *p*-value < 0.10. The significant explanatory variables contained in the separate models will then be combined and re-examined using further modeling. We plan to use multiple linear regression to assess which of the included explanatory variables and/or their interactions with PA change predict change in RMDQ. For those variables which remain in the final model, an examination of their significance (p < 0.05) will be undertaken to evaluate their contribution to the final model.

At 1 year the relationship between ΔRT3 and presence or absence of on-going low back pain (Y/N) from the modified Nordic LBP questionnaire will be investigated using logistic regression. At 1 year the relationship between the BPAQ change score from baseline (pre-LBP) to BPAQ score at 1 year (ΔBPAQ) with the outcome of on-going LBP (Y/N) will also be explored using logistic regression.

## Discussion

There is also a need to evaluate cost-effective methods to mange LBP within the community [83] and to provide objective evidence for the role of activity in the management of LBP [84]. This research will investigate whether relationships exist between objectively measured PA and functional recovery in a LBP population. Evidence for such effects will also allow assessment of the relationships between LBP recovery and reoccurrence at 1 year, occupational activity, and objectively measured free living activity. Such results will add to our knowledge on the relationships between the amount of, and changes in, free living activity relative to LBP recovery and might be an important step for determining future application of activity prescription and the potential use of activity monitoring in the management of primary care LBP.

It must also be acknowledged that this specific cohort, collected from physiotherapy practices, may differ in its activity levels; its view of activity and the advice it is given regarding activity when compared to all other community dwelling populations with acute LBP. It is therefore planned to assess the generalisability of the results from this study by comparing this cohort to other primary care cohorts investigating acute LBP within Australasia

## Abbreviations

ACC: Accident Compensation Corporation; BPAQ: Baecke Physical Activity Questionnaire Fear-Avoidance Beliefs Questionnaire; GHQ12: 12-item General Health Questionnaire; LBP: low back pain; PA: physical activity; PAEE: physical activity energy expenditure; PI: principal investigator; RMDQ: Roland Morris Disability Questionnaire; VAS: Visual Analogue Scale; FABQ: TDEE: total daily energy expenditure; VM: Vector magnitude: ΔBPAQ: change in Baecke Physical Activity Questionnaire; ΔRT3: change in RT3 VM: ΔRMDQ: change in Roland Morris Disability Questionnaire score; 7D-PAR: 7-Day Recall Questionnaire.

## Competing interests

The authors declare that they have no competing interests.

## Authors' contributions

PH is the principal investigator. PH together with his supervisory team of SM, MB, LH and DB designed the study and were responsible for the protocol. SMc and DH acted as international advisors and helped in the development of key ideas underlying this study. MB is responsible for the sample size and power calculation, for the design of the statistical analysis and the evaluation of the database. MM helped in the study design and all authors read and approved the final manuscript.

## Pre-publication history

The pre-publication history for this paper can be accessed here:


